# Different fat-to-fiber ratios by changing wheat inclusion level impact energy metabolism and microbial structure of broilers

**DOI:** 10.3389/fmicb.2024.1298262

**Published:** 2024-03-19

**Authors:** Qiuyu Jiang, Lihua Zhao, Zhibin Ban, Bingkun Zhang

**Affiliations:** ^1^State Key Laboratory of Animal Nutrition and Feeding, College of Animal Science and Technology, China Agricultural University, Beijing, China; ^2^Laboratory of Animal Nutrition Metabolism, Jilin Academy of Agricultural Sciences, Jilin, China

**Keywords:** energy, fat, non-starch polysaccharide, microbiota, broiler

## Abstract

**Introduction:**

Dietary nutrient content is crucial for energy metabolism and development of gut microbiota. Herein, this study aimed to explore the effects of fat-to-fiber ratios on nutrient transporter, energy metabolism and gut microbiota when ingredients composition was altered.

**Methods:**

A total of 240 as-hatched broiler chickens were randomly assigned into three groups including low fat-high dietary fiber (LF-HD), medium fat-medium dietary fiber (MF-MD) and high fat-low dietary fiber (HF-LD), with diets being iso-protein, and broilers were offered the same commercial diets from 21 to 42 d. The data were analyzed using one-way ANOVA of SPSS.

**Results and Discussion:**

Results showed that HF-LD diet significantly increased glucose content and decreased triglyceride in serum of broilers (*p* < 0.05). The mRNA abundance of jejunal gene involved in glucose transporter and tricarboxylic acid (TCA) cycle was significantly increased in broilers fed with HF-LD diets. Compared with LF-HD, HF-LD had a lower abundance of *Anaerofilum* and *CHKCI001*, and an increased proportion of beneficial bacteria such as *Alistipes*, *Catenibacillus*, *Intestinimonas*, *Lactobacillus*, and *Peptococcus* (*p* < 0.05). Functional prediction of these microbial changes indicated that HF-LD diet drove caecal microbiota to participate in carbohydrate metabolism and TCA cycle (*p* < 0.05). Dietary HF-LD-induced microbiota changes were positively correlated with growth performance of broilers (*p* < 0.05). Therefore, HF-LD diet increased glucose transporters and energy metabolism in intestine and shaped microbial structure and metabolic pathways, which may benefit the growth performance of broilers.

## Introduction

Corn is the main energy source in diets fed to mono-gastric animal. Due to the imbalance of supply and demand of corn in the poultry industry, wheat has been increasingly used in the diets. However, wheat contains non-starch polysaccharide (NSP) about 50% higher than corn, which may encapsulate other nutrients and increase the viscosity of digesta (Zhang T. et al., 2018; Pan and An, 2020). In fact, energy derives from a wheat-based diet is less readily available than that from a corn-based diet, attributed largely to the presence of soluble NSP content, especially for arabinoxylans (Theander et al., 1994; Nguyen et al., 2022). Therefore, increasing wheat content in the diet could be accompanied by an increase in fiber content. Fiber-rich wheat and corn differently impact nutrient utilization (Chen et al., 2019), the composition of microbiota (Ghiasvand et al., 2021) and growth performance (Schoenherr et al., 1989) of broilers.

Dietary energy density and nutrient composition, especially fat-to-fiber ratios, play major roles in regulating nutrient and energy utilization of animals (Barekatain et al., 2021). On the one hand, fat produces less heat energy loss in the process of digestion and absorption in the intestine. It was recognized that dietary fat and fiber differently affect heat increment (HI) and net energy to metabolizable energy ratio (fat > starch >CP = fiber) of monogastric animals (Noblet et al., 1994a; Wu et al., 2019). Reducing fiber and increasing fat content in the diet increased energy intake of broilers (Latshaw, 2008) On the other hand, fat and fiber have different characteristics for digestion and absorption in the intestine. Fat reduces the passage rate of digesta through gut tract, allowing for better nutrient and energy utilization (Jiménez-Moreno et al., 2009). The NSP is incapable of depolymerize in the small intestine and is mainly hydrolyzed in the hindgut via microbial fermentation (Romero et al., 2014; Kim et al., 2022). Therefore, the energy efficiency of digestion and fermentation of fiber-rich diet could be different from the digestion and absorption of fat.

The gastrointestinal tract constitutes up to 23% ~ 36% of total body energy expenditure (Hunter and Mitchell, 2002). The intestine is an organ highly dependent on energy for digestion, absorption and renewal of epithelium. Dietary nutrients could be sensed and regulated by intestine to meet the energy requirement (Dyer et al., 2007). These absorbed nutrients in the small intestine experienced a series of metabolic pathway such as tricarboxylic acid (TCA) and oxidative phosphorylation to produce biological energy. There are literatures illustrated that the low energy intake could decreased the energy status in the small intestine (Wang et al., 2022). Nutritional strategies have been extensively documented to improve energy metabolism in the intestine (Pi et al., 2014; Zhang H. et al., 2019). However, to our knowledge, this is the first attempt to explore the effects of dietary energy density especially fat-to-fiber ratio on intestinal energy metabolism.

Diet composition and energy density are powerful factors affecting gut health and shaping gut microbiota (Wang et al., 2015; Adewole and Akinyemi, 2021). As is all known, fiber-rich diets could alter the microbial richness and diversity in the hindgut. The microbial fermentation produces energy substances such as short-chain fatty acid and bile acid. Previous studies reported that SCFA, especially for butyrate was positively correlated with intestinal energy metabolism (Li et al., 2019), epithelium integrity and mucosal growth (Wu et al., 2021; Cantoni et al., 2022). Moreover, during the fermentation process, microbiota provides intermediates and enzymes that were related to metabolism of carbohydrate, lipid and amino acid (Zhang Y. et al., 2019). Dietary nutrient shifts the microbial composition and metabolic pathway of microbiota, which may further affect nutrient utilization and hindgut health. Although evidence has demonstrated the effects of fiber on intestinal microbiota, the microbial changes in response to different fat-to-fiber ratios are still unknown. The hypothesis of this study is that an increase in the dietary fat-to-fiber ratios could improve the energy metabolism and alter the composition and function of gut microbiota, thereby supporting intestinal health and overall growth performance. Herein, the effects of dietary fat-to-fiber ratios on growth performance, energy metabolism, composition and function of gut microbiota in broilers are determined.

## Materials and methods

### Experiment diets

The experiment comprised a 2-phase feeding program including a starter phase (from hatch to 21 d) and a finisher phase (from 21 to 42 d). Broilers were administrated three diets at starter phase: low fat-high dietary fiber (LF-HD), medium fat-medium dietary fiber (MF-MD) and high fat-low dietary fiber (HF-LD). Different diets were formulated by changing the fiber-rich wheat (neutral detergent fiber, NDF, 14.6%) with corn and increasing the additional level of soybean oil with a maximum level of 4.20%. The fat content gradually increased (3.88, 4.86, and 7.55%, respectively) and total dietary fiber content gradually decreased (6.73, 3.57, and 2.06%, respectively) from LF-HD to HF-LD diets. Dietary fat-to-fiber ratios were changed accordingly (0.58, 1.36, 3.67) from LF-HD to HF-LD diets. As shown in Table 1, diets were formulated to exceed the nutrient and energy requirement as recommended by NRC (1994). All experiment diets were pelleted in a similar way. A commercial diet (from New Hope Liuhe Co., Ltd) for broilers at the finisher phase was adopted to explore the persistent effects of fat and fiber content on broilers.

**Table 1:** Ingredients and nutrient contents of experiment diets of broilers from 1 to 21 d (as-fed basis)^1^.

Items	LF-HD	MF-MD	HF-LD
Ingredients, %			
Wheat (NDF, 14.6%)	69.67	54.00	17.00
Corn	0.00	14.45	47.49
Soybean oil	1.99	2.55	4.20
Protein mix^2^	23.45	24.17	26.65
Dicalcium phosphate	1.80	1.80	1.80
Limestone	1.30	1.30	1.30
Choline chloride	0.30	0.30	0.30
DL-Methionine	0.08	0.09	0.09
L-Lysine	0.84	0.77	0.60
Salt	0.35	0.35	0.35
Vitamin premix^3^	0.02	0.02	0.02
Trace minerals premix^4^	0.20	0.20	0.20
Total	100	100	100
Chemical composition, %			
Metabolizable energy, kcal/kg	2,965	2,964	2,969
NE/AME^5^	74.60	74.86	75.67
Crude protein	21.07	21.17	21.00
Calcium	1.00	0.98	0.95
Available phosphorus	0.43	0.42	0.40
Lysine	1.19	1.22	1.29
Methionine	0.46	0.46	0.44
Threonine	0.57	0.61	0.71
Insoluble fiber	5.88	3.11	1.78
Soluble fiber	0.85	0.46	0.27
Lignin	0.80	0.71	0.48
Pectins	0.71	0.66	0.42
NSP	9.72	8.68	5.98
Insoluble NSP	8.69	8.02	6.15
Soluble NSP	1.90	1.48	0.47
Analyzed value, %			
Gross energy, kcal/kg	4,109	4,126	4,181
Crude protein	21.57	21.57	21.11
Crude fat	3.88	4.86	7.55
Total dietary fiber	6.73	3.57	2.06
Fat-to-fiber ratio	0.58	1.36	3.67
NDF	14.29	13.64	12.26
ADF	4.31	3.09	2.61

1 LF-HD, low fat-high dietary fiber; MF-MD, medium fat-medium dietary fiber; HF-LD, high fat-low dietary fiber; NDF, neutral detergent fiber; ADF, acid detergent fiber; NSP, non-starch polysaccharide; NE, net energy; AME, apparent metabolizable energy.

2 Protein mixture contains soybean meal, degossypolled cottonseed meal and corn gluten meal to maintain a consistent protein content of diets.

3 Vitamin minerals premix provided the following per kg of diets: Vitamin A 9, 500 IU, Vitamin D 362.5 μg, Vitamin E 30 IU, Vitamin K3 2.65 mg, Vitamin B1 2 mg, Vitamin B2 6 mg, Vitamin B6 6 mg, Vitamin B12 0.025 mg, biotin 0.0325 mg, folic acid 1.25 mg, pantothenic acid 12 mg, nicotinic acid 50 mg.

4 Trace minerals premix provided the following per kg of diets: Cu 8 mg, Zn 75 mg, Fe 80 mg, Mn 100 mg, Se 0.15 mg, I 0.35 mg.

5 NE/AME is calculated by dietary crude protein and fat content using equations (NE/ME = 79.2–0.26*CP + 0.26*EE) established by Wu et al. (2019).

### Birds management and experiment design

The animal experiment was approved by Institutional Animal Care and Use Committee, China Agricultural University. A total of 240 as-hatched Arbor Acres broilers with similar body weights were enrolled in the study and reared in animal facilities (Zhuozhou, Hebei, China). The husbandry practices (including immunization procedure, humidity, temperature and light program) were undertaken according to the Arbor Acres broilers management guidelines with minor modifications to suit the experimental conditions. Birds were randomly allocated into 3 groups with 8 replicates per group, and 10 birds per replicate. Following this, all broilers were exposed to their respective diets with free access to water and feeds. After 12 h starving at 21 d, one bird from each replicate was sacrificed by electrical stunning for sample collection.

### Chemical analysis

The gross energy (GE) of diets was determined using a bomb calorimeter (IKA-C3000, Germany), standardized with benzoic acid. Crude protein was determined by the Kjeldahl method (AOAC, 990.03) using semi-automated Kjeldahl distillation (KT 200 Kjeltec distillation unit, Hilloeroed, Denmark). Crude fat content was assayed using the method of AOAC (920.39). The NDF and acid detergent fiber (ADF) of diets were determined using a fiber analyzer (ANKOM-A2000i, America) following a procedure by Van Soest et al. (1991). Total dietary fiber was determined by using method AOAC 985.29. Basically, dietary fiber was determined by enzymatic hydrolysis and followed by ethanol precipitation.

### Serum and sample preparation

After fasting for 12 h, one bird per replicate with averaged body weight was electrocuted. Before euthanasia, the blood was drawn from the jugular vein and then centrifuged at 3,000 × *g* at 4°C for 15 min to obtain serum. Immediately after euthanasia, about 2 cm tissue of mid-jejunum was collected. The tissues were washed in phosphate buffer saline and then frozen in liquid nitrogen until RNA extraction. The cecum contents were collected for microbiota analysis.

### Serum metabolites

Serum metabolites including glucose (GLU), fatty acid (FFA), low-density lipoprotein (LDL), high-density lipoprotein (HDL) and triglyceride (TG) were measured using an automatic biochemical analyzer (iCubio Chemistry Analyzer, iChem 340, Shen Zhen, China).

### Total RNA extraction and real-time qPCR

Approximately 1 g of jejunal tissues were cut from the frozen sample and immediately homogenized in 1 mL Trizol reagents (Genstar, China) to extract total RNA following the manufacturer’s protocol. The concentration and purity of extracted RNA were verified at 260/280/230 nm by Nanodrop 2000 spectrophotometer (Thermo Scientific, Waltham, MA). Then cDNA was synthesized from 1,000 ng total RNA following the manufacturer’s protocol with High Capacity cDNA Reverse Transcription Kit (Genstar, China). Real-time qPCR was run on a Step Two Plus System (Thermo Fischer), using Genstar RT-PCR kit according to the manufacturer’s protocol. Reactions were performed following a previous study (Zhang S. et al., 2018). Table 2 displays the sequence of primers that were used to amplify target genes. The target gene expression normalized by *β-actin* was calculated and analyzed by the 2^−ΔΔCt^ method.

**Table 2:** Primer sequences used in the real-time qPCR.

Item	Primer sequence (5′ to 3′)	GenBank accession No.
*ATP5B*	F: AAGGCTCCATCACTTCGGTG	NM_001031391.3
	R: TGTTGGGGTCCATGATTCGG	
*OGDH*	F: TTCAAGCACAGCCCAACGTA	NM_001031382.2
	R: GCCCGTAAAATCCGACGTTT	
*SDHA*	F: ATTCCCGTTTTGCCTACGGT	NM_001277398.1
	R: GGGAGTTTGCTCCAAGACGA	
*SDHB*	F: GGTCCAGGGGATCTGTCG	NM_001080875.3
	R: GTTCATTGCACAGGAGCCAC	
*SUCLG1*	F: TGACCTTCCAGGACAACTGA	NM_001012892.2
	R: GGAACGCCTCCTCAACGTAA	
*SUCLG2*	F: ACTTCCTGGATCTGGGTGGA	NM_001006141.2
	R: CTCTCGGCAGGCTTTGGTAA	
*SGLT1*	F: CCCTTCCAACTGTCCGTTCA	NM_001293240
	R: CCAGCACAAGCGATAAAGATGTA	
*GLUT5*	F: CATCTTCTTCATTGTTCCTGAGAC	XM_417596
	R: CAAATCCATCATCTGTTCCACA	
*FABP1*	F: CACCATTGGGGAAGAGTGTGA	NM_204192.4
	R: GTTCGGTCACGGATTTCAGC	
*FABP2*	F: TGGAAGCAATGGGCGTGAAT	NM_001007923.1
	R: TGTCGATGGTACGGAAGTTGC	
*FATP4*	F: AATGATGGAAGCTATGAAGGAGGT	XM_015279553.4
	R: TCATCGGGTCTCATGCGGAA	
*β-actin*	F: CAACACAGTGCTGTCTGGTGGTAC	XM_027015741.1
	R: CTCCTGCTTGCTGATCCACATCTG	

*ATP5B*, recombinant ATPase, H+ transporting, mitochondrial F1 complex β; *OGDH*, oxoglutarate dehydrogenase; *SDHA*, succinate dehydrogenase A; *SDHB*, succinate dehydrogenase B; *SUCLG1*, succinate-CoA ligase GDP/ADP-forming subunit α; *SUCLG2*, succinate-CoA ligase GDP/ADP-forming subunit β; *SGLT1*, sodium dependent glucose transporters 1; *GLUT5*, facilitated glucose transporter 5; *FABP1*, fatty acid binding protein 1; *FABP2*, fatty acid binding protein 2; *FATP4*, fatty acid transport protein 4. F, forward primer; R, reverse primer.

### DNA extraction and 16S RNA gene sequencing

Total bacterial DNA was extracted from caecal samples and the quality of DNA was assessed by 1% agarose gel electrophoresis. The V3-V4 hypervariable regions of bacterial 16 rRNA gene were amplified with primers 338F (5′-ACTCCTACGG GAGGCAGCAG-3′) and 806R (5′-GGACTACHVGGG TWTCTAAT-3′) by using thermal cycling PCR system (GeneAmp 9700, ABI, USA). PCR conducts were extracted from 2% agarose gel and then purified by AxyPrep DNA Gel Extraction Kit (Axygen Biosciences, USA) and quantified by QuantiFluor™ – ST blue fluorescence quantitative system (Promega, USA). At last, the library for the samples were pooled at equal ratio and sequenced on the Illumina MiSeq platform. Raw sequencing reads could be found in the NCBI Sequence Read Archive (SRA) under BioProject PRJNA952536.

### Sequencing data analysis and functional prediction

Paired-end reads sourced from the original DNA fragments were merged into tags using FLASH (version 1.2.11).^1^ Sequencing process was performed by using UPARSE software package (version 11)^2^ with the UPARSE-OUT algorithms. MOTHUR (1.30.2)^3^ and QIIME (version 1.9.1)^4^ were used to assess alpha diversity within samples and beta diversity among samples. Sequences were assigned to the same operational taxonomic units (OTUs) when the similarity reaches 97%. Functional prediction was performed by using PICRUSt (version 1.1.0).^5^ The difference in metabolic pathways were analyzed using KEGG pathway search function.

### Statistical analysis

Each pen was considered as the experimental unit for the performance outcome parameters. Serum metabolites, jejunal gene expression, relative abundance of differential microbiota and functional metabolites in 3 groups were analyzed using One-way ANOVA followed by Duncan’s multiple range tests using the GLM procedure of SPSS (version 16.0, SPSS Inc., Chicago, IL, USA). Mean and pooled standard errors of the mean (SEM) were calculated for each variable. Differences were accepted as representing statistically significant differences when *p* < 0.05. ANOSIM was performed based on Bray–Curtis dissimilarity to determine distance between three groups of microbial communities. The bacterial variation and the metabolic pathway were analyzed using the linear discriminant analysis (LDA) of effect size (LEfSe) biomarker discovery tool (*p* < 0.05 and LDA score > 2.5). Moreover, spearman correlation analysis was carried out to estimate relationships between microbiota at genus level and growth phenotypes of broilers.

## Results

### Serum metabolites

**Figure 1:**
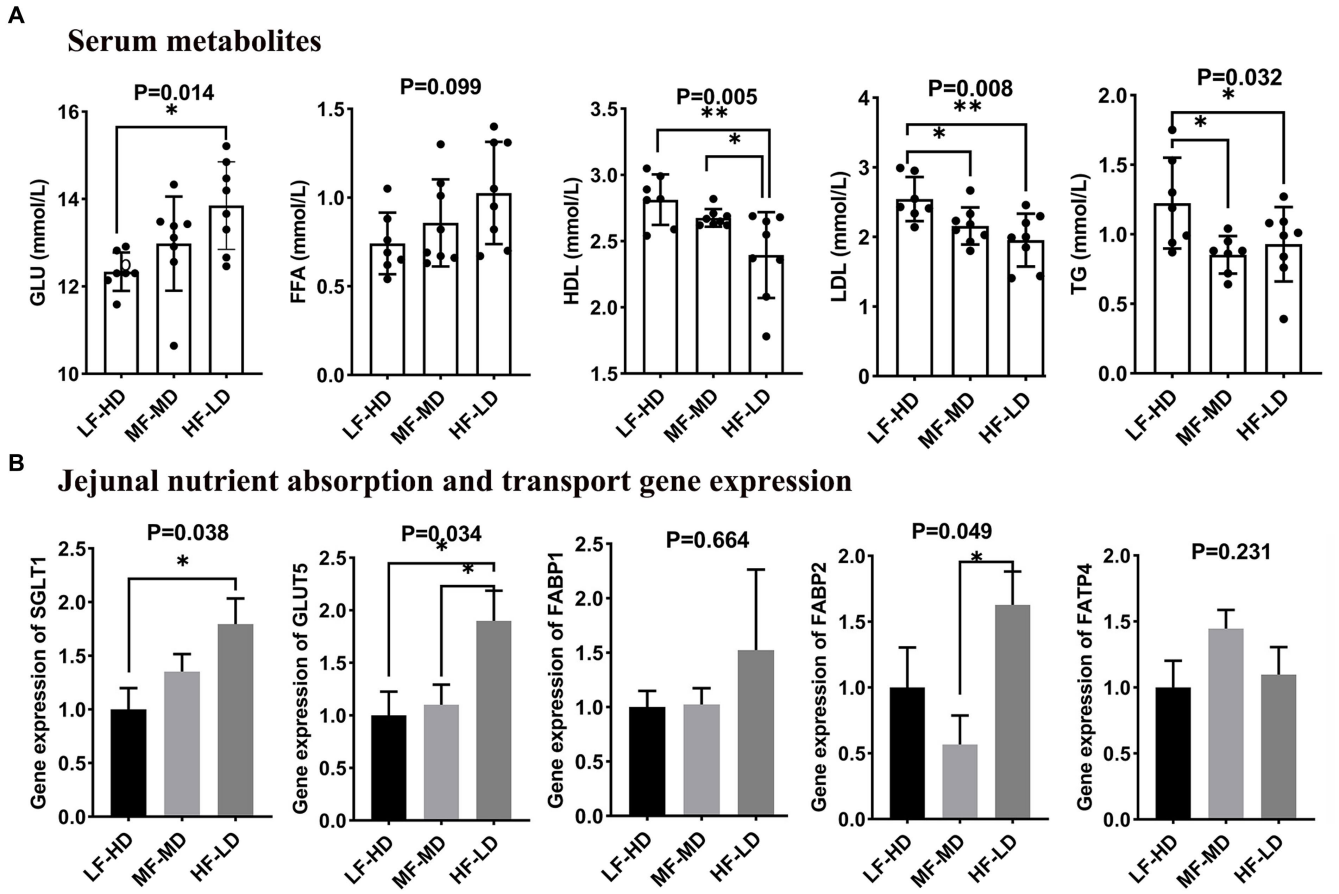
Effects of fat-to-fiber ratio on serum metabolites **(A)** and jejunal gene expression related to nutrient transport **(B)** of broilers. GLU, glucose; FFA, free fatty acid; LDL, low-density lipoprotein; HDL, high-density lipoprotein; TG, triglyceride; *SGLT1*, sodium dependent glucose transporters 1; *GLUT5*, glucose transporter 5; *FABP1*, fatty acid binding protein 1; *FABP2*, fatty acid binding protein 2; *FATP4*, Fatty acid transport protein 4; LF-HD, low fat-high dietary fiber; MF-MD, medium fat-medium dietary fiber; HF-LD, high fat-low dietary fiber. Significant difference was recorded by *p < 0.05, **p < 0.01, *n* = 8.

The effects of dietary composition and fat-to-fiber ratios on serum metabolites of 21-day-old broilers that related to lipid and glucose metabolisms are shown in Figure 1A. Diet HF-LD significantly increased glucose content in serum compared with LF-HD diet (*p* < 0.05). Moreover, the LDL and TG levels significantly declined in the serum of birds fed with MF-MD and HF-LD diets relative to birds fed with LF-HD diet (*p* < 0.05). Compared with LF-HD and MF-MD diets, HF-LD diet significantly decreased HDL content in serum of broilers at 21 d (*p* < 0.01). In addition, FFA content was numerically higher in birds fed with HF-LD diet (*p* = 0.099).

### Jejunal gene expression related to energy metabolism

Table 3 displayed jejunal gene expression of enzymes that participate in TCA cycle of 21-day-old broilers. The oxoglutarate dehydrogenase (*OGDH*) and succinate dehydrogenase (*SDHB*) levels of HF-LD group increased considerably relative to other groups (*p* < 0.05). Relative to HF-LD diet, the decreased gene expression of succinyl-coA ligase GDP-forming β subunit (*SUCLG2*) in the jejunum was only notable upon MF-MD diet (*p* < 0.01).

**Table 3:** Effects of fat-to-fiber ratio^1^ on metabolic markers related to energy metabolism^2^.

Items^3^	LF-HD	MF-MD	HF-LD	SEM^4^	*p*-value
*ATP5B*	1.00	1.18	1.34	0.142	0.643
*OGDH*	1.00^b^	1.02^b^	2.02^a^	0.134	<0.001
*SDHA*	1.00	0.65	0.74	0.076	0.144
*SDHB*	1.00^b^	0.93^b^	1.43^a^	0.088	0.035
*SUCLG1*	1.00	0.72	0.99	0.066	0.144
*SUCLG2*	1.00^a^	0.51^b^	1.22^a^	0.081	<0.001

1 LF-HD, low fat-high dietary fiber; MF-MD, medium fat-medium dietary fiber; HF-LD, high fat-low dietary fiber.

2 Each mean represents values from 8 replicates (birds).

3 *ATP5B*, recombinant ATPase, H+ transporting, mitochondrial F1 complex β; *OGDH*, oxoglutarate dehydrogenase; *SDHA*, succinate dehydrogenase A; *SDHB*, succinate dehydrogenase B; *SUCLG1*, succinate-CoA ligase GDP/ADP-forming subunit α; *SUCLG2*, succinate-CoA ligase GDP/ADP-forming subunit β.

4 SEM, standard error of mean.

^a,b^Means within rows with different superscripts are significantly different (*p* < 0.05, *n* = 8).

### Jejunal gene expression related to nutrient transport

As illustrated in Figure 1B, a significant increase of glucose transporter 5 (*GLUT5*) in jejunal was observed in birds fed HF-LD diet compared with other diets (*p* < 0.05). Besides, the jejunal gene expression of sodium-dependent glucose transporters 1 (*SGLT1*) was up-regulated in birds fed with HF-LD diet compared with those fed LF-HD diet (*p* < 0.05). Birds fed HF-LD diet exhibited relatively higher gene expression of fatty acid binding protein 2 (*FABP2*) in jejunum relative to those fed MF-MD diet (*p* < 0.05), whereas there was no difference relative to birds fed with LF-HD diet (*p* > 0.05).

### Cecum microbial diversity

**Figure 2:**
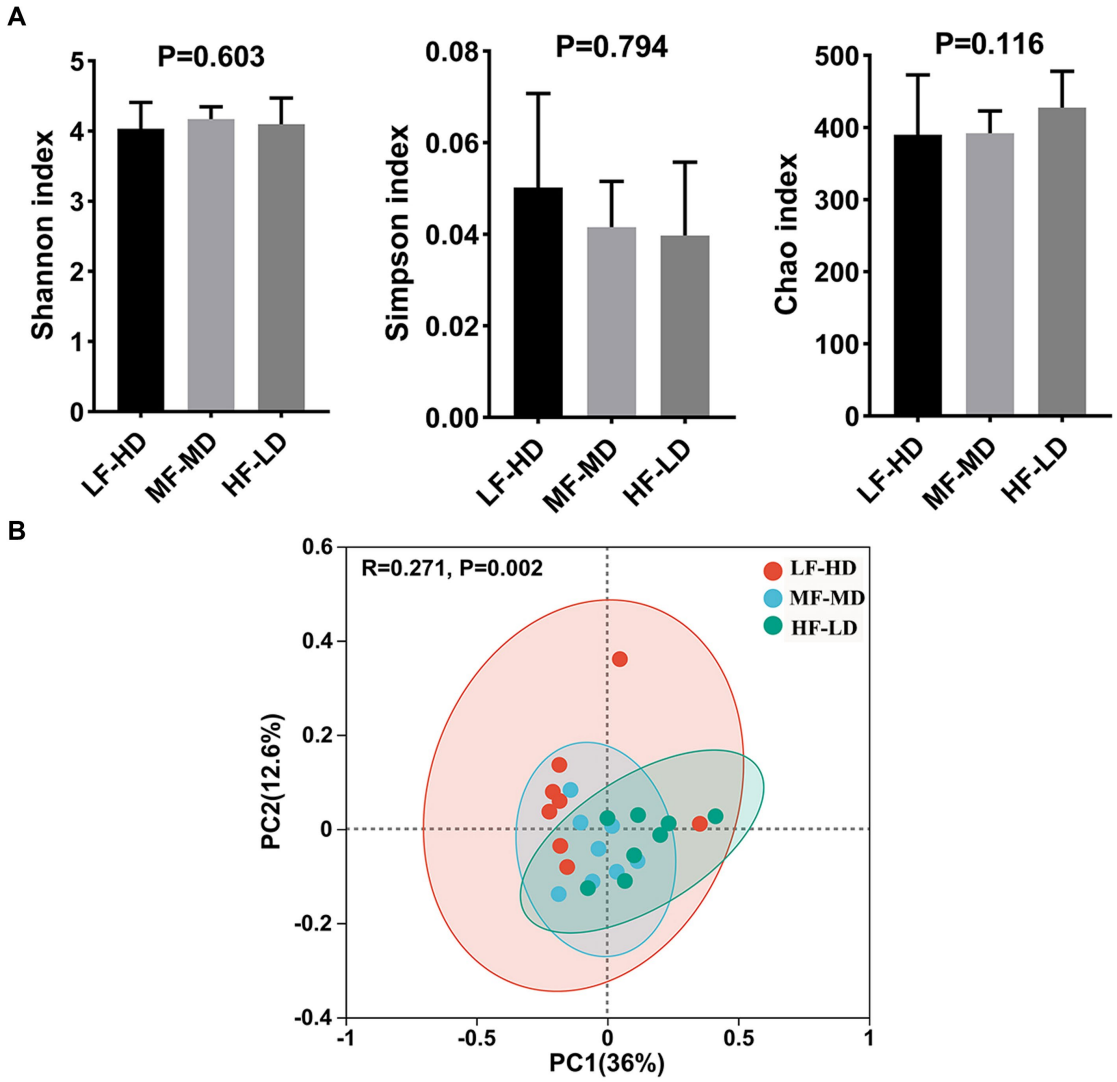
Alpha and beta diversity of caecal microbial communities of broilers at 21 d fed diets with different fat-to-fiber ratio. **(A)** Alpha diversity of three index including Shannon, Simpson and Chao. **(B)** Beta diversity of caecal microbiota communities was calculated based on bray curtis distance for principal coordinate analysis (PCoA). LF-HD, low fat-high dietary fiber; MF-MD, medium fat-medium dietary fiber; HF-LD, high fat-low dietary fiber. Significant difference was recorded by *p < 0.05, **p < 0.01, *n* = 8.

*Alpha*-diversity and *Beta*-diversity indices, estimating the bacterial diversity and richness of caecal digesta of 21-day-old broilers, are shown in Figure 2. Shannon, Simpson and Chao index had no significant difference in these groups (*p* > 0.05). The scatter plot of principal coordinate analysis (PCoA) displayed significant separation (*p* = 0.002) of caecal microbiota in three groups.

### Composition of caecal microbiota

**Figure 3:**
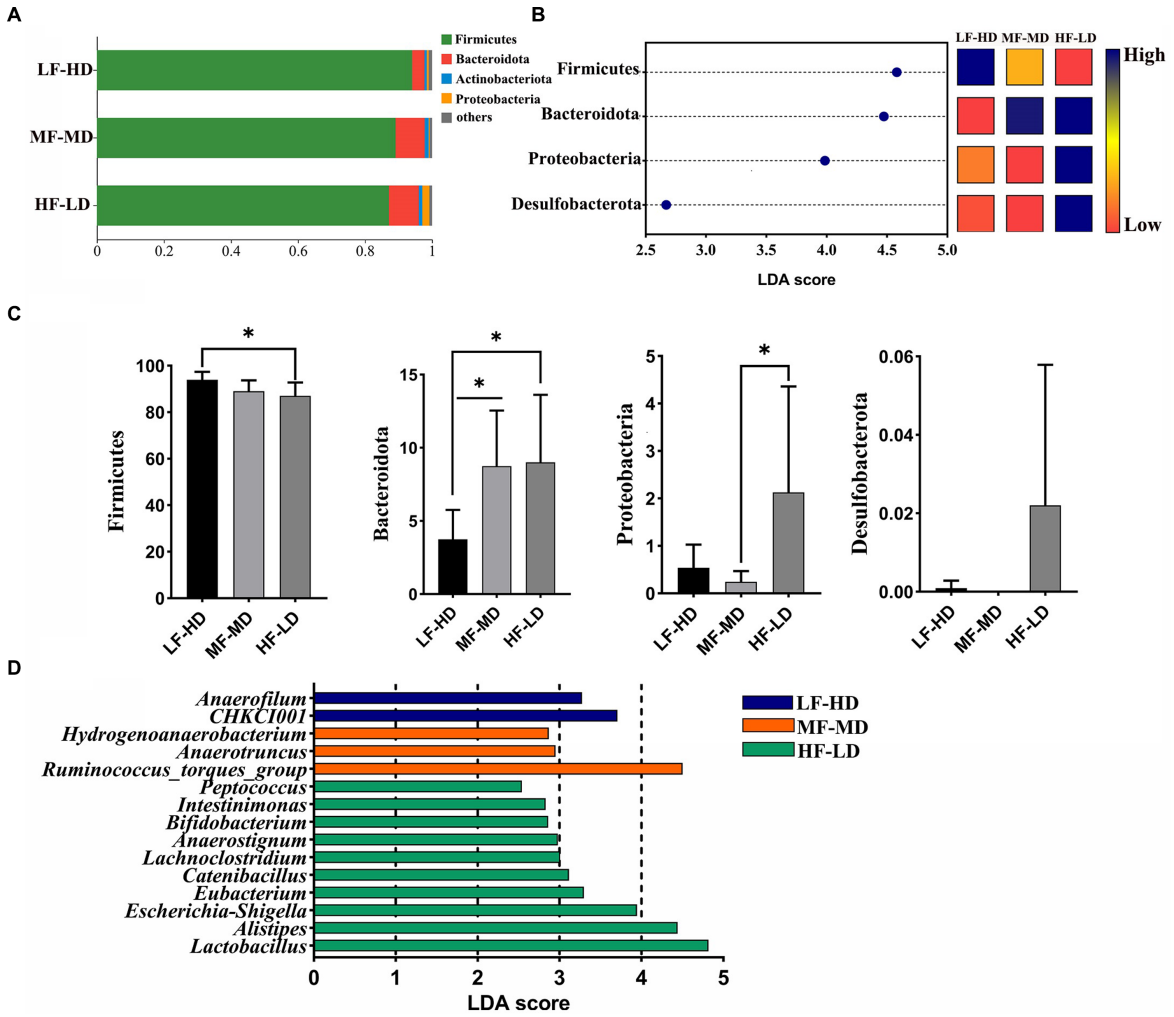
Effects of fat-to-fiber ratio on caecal microbiota composition of 21 d broilers. **(A)** The relative abundance of microbiota at phylum level. **(B)** The differentially abundant microbiota at phylum level by using LDA scores (*p* < 0.05 and LDA score > 2.5). Heatmap shows the relative abundance of microbiota at phylum level. The blue represents high abundance of microbiota while the red represents low abundance of microbiota. **(C)** The differentially abundant microbiota at phylum level. **(D)** The differentially abundant microbiota at genus level using linear discriminant analysis (LDA) scores (*p* < 0.05 and LDA score > 2.5). LF-HD, low fat-high dietary fiber; MF-MD, medium fat-medium dietary fiber; HF-LD, high fat-low dietary fiber. Significant difference was recorded by *p < 0.05, *n* = 8.

Figure 3A exhibits the composition of caecal microbiota. Firmicutes were the most dominant phylum microbiota in the cecum, then Bacteroidota ranked second, followed by the Actinobacteriota and Proteobacteria. As shown in Figures 3B,C, the relative abundance of Bacteroidota experienced a distinct decline in LF-HD diet compared with other diets (*p* < 0.05). However, Firmicutes significantly increased in LF-HD diet compared with HF-LD diet. In addition, there was a higher abundance of Proteobacteria in HF-LD diet compared to MF-MD diet (*p* < 0.05). Figure 3D displayed the relative abundance of microbiota at genus level. The abundance of *Anaerofilum* and *CHKCI001* significantly elevated (*p* < 0.05) in LF-HD diet, while the abundance of *Peptococcus*, *Intestinimonas*, *Bifidobacterium*, *Anaerostignum*, *Lachnoclostridium*, *Catenibacillus*, *Eubacterium*, *Escherichia-Shigella*, *Alistipes* and *Lactobacillus* significantly increased in HF-LD diet (*p* < 0.05). Besides, *Hydrogenoanaerobacterium, Anaerotruncus* and *Ruminococcus*_torgues_group significantly increased upon MF-MD diet (*p* < 0.05).

### Functional prediction of microbiota

**Figure 4:**
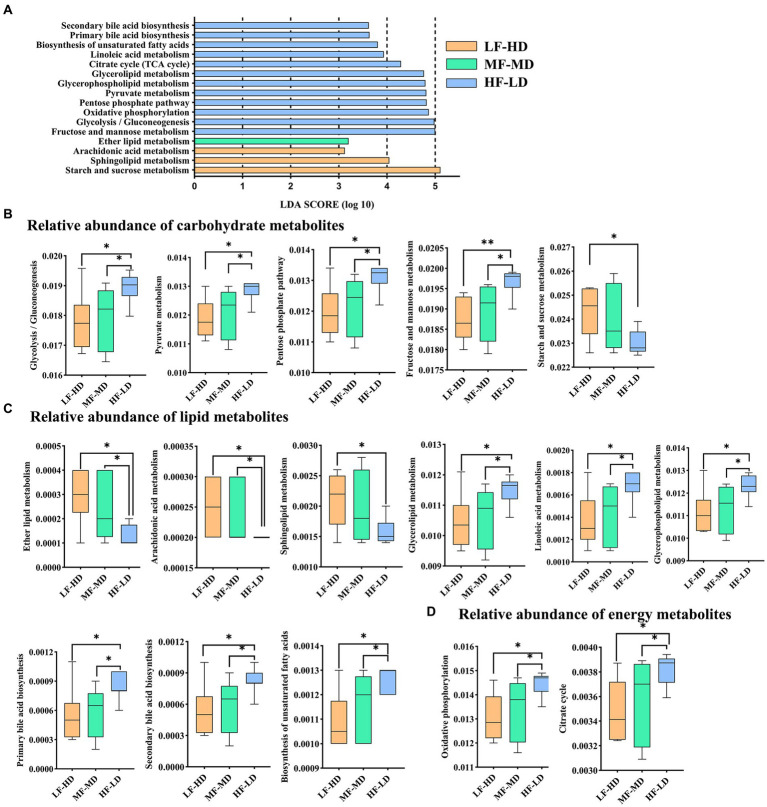
Effects of fat-to-fiber ratio on metabolic pathways of caecal microbiota of 21 d broilers. **(A)** The differentially abundant metabolic pathways using LDA scores (*p* < 0.05 and LDA score > 2.5). **(B)** Relative abundance of carbohydrate metabolites. **(C)** Relative abundance of lipid metabolites. **(D)** Relative abundance of energy metabolites. LF-HD, low fat-high dietary fiber; MF-MD, medium fat-medium dietary fiber; HF-LD, high fat-low dietary fiber. Significant difference was recorded by *p < 0.05, **p < 0.01, *n* = 8.

As shown in Figure 4A, 16 metabolic pathways of KEGG involved in carbohydrate (Figure 4B), lipid (Figure 4C) and energy (Figure 4D) metabolisms were identified between three groups. HF-LD group enriched carbohydrate metabolism including glycolysis/gluconeogenesis, fructose and mannose, pentose phosphate and pyruvate metabolism compared with other groups (*p* < 0.05), but depleted starch and sucrose metabolism compared with LF-HD group (*p* < 0.05). Additionally, HF-LD group increased lipid metabolism including linoleic acid, biosynthesis of unsaturated fatty acids, glycerolipid, glycerophospholipid and bile acid biosynthesis compared with other groups (*p* < 0.05). Interestingly, lipid metabolism of ether lipid and arachidonic acid decreased markedly in HF-LD group compared with other groups (*p* < 0.05), while sphingolipid metabolism decreased in HF-LD group compared with LF-HD group (*p* < 0.05). Moreover, Oxidative phosphorylation and citrate cycle metabolism significantly improved in HF-LD group compared with other groups (*p* < 0.05).

### Correlation analysis between microbiota and growth phenotypes

**Figure 5:**
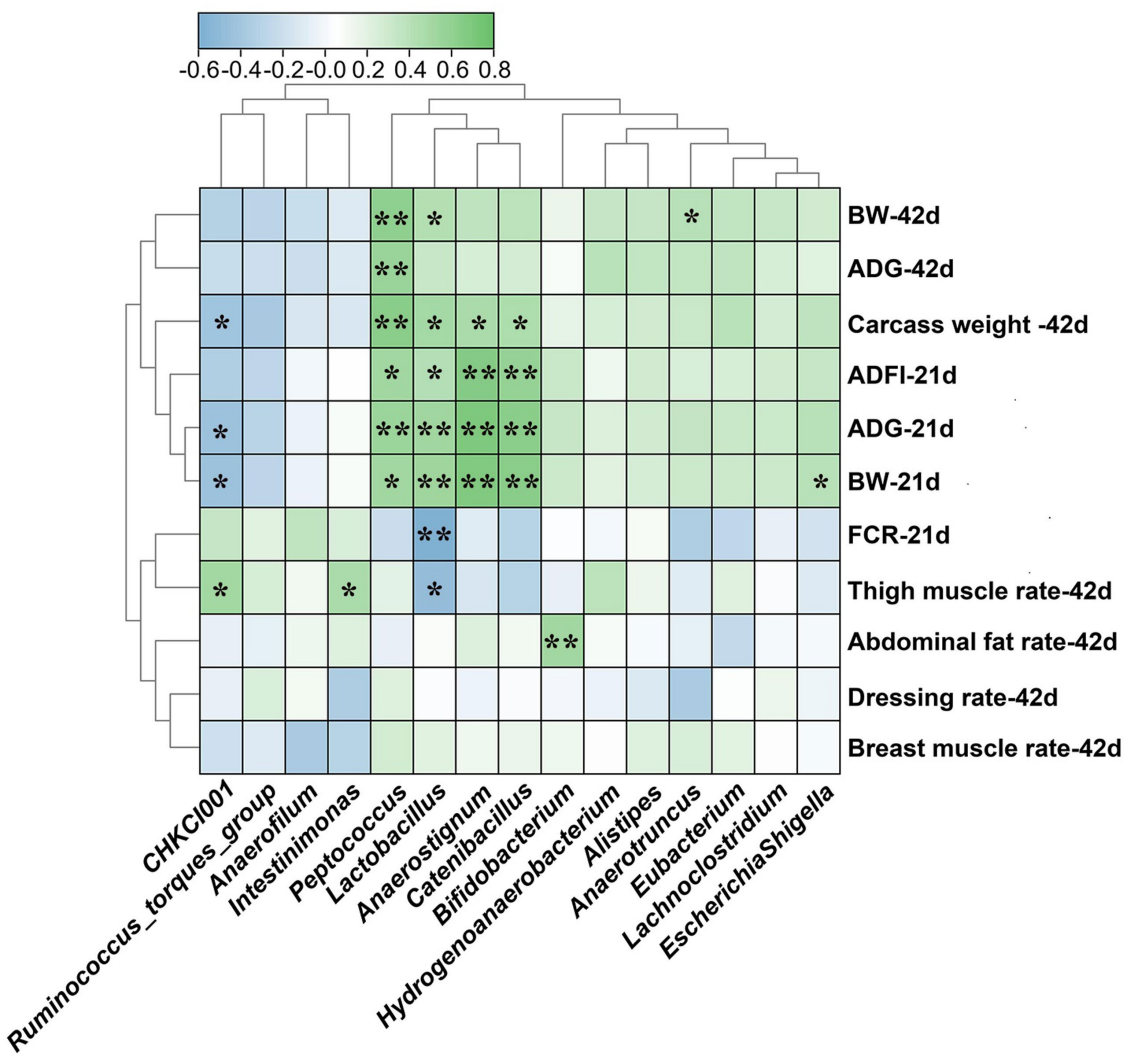
Spearman correlations between differential caecal microbiota at genus level and growth performance of broilers. BW, body weight; ADG, average daily gain; ADFI, average daily feed intake; FCR, feed conversion ratio. LF-HD, low fat-high dietary fiber; MF-MD, medium fat-medium dietary fiber; HF-LD, high fat-low dietary fiber. Significant difference was recorded by *p < 0.05, **p < 0.01, *n* = 8.

Spearman correlation analysis was carried out to estimate relationships between microbiota at genus level and growth phenotypes of broilers (Figure 5). The abundance of *Peptococcus*, *Lactobacillus*, *Anaerostignum* and *Catenibacillus* were positively correlated with BW, ADG, ADFI of 21 d broilers and carcass weight of 42 d broilers (*p* < 0.05). Additionally, the abundance of *Lactobacillus* was positively correlated with BW of 42 d broilers but negatively correlated with FCR of 21 d broilers and thigh muscle rate of 42 d broilers (*p* < 0.05). The *Peptococcus* was positively correlated with BW and ADG of 42 d broilers (*p* < 0.05). The abundance of *CHKCI001* was positively correlated with thigh muscle rate of 42 d broilers (*p* < 0.05) but negatively correlated with ADG, BW of 21 d broilers and carcass weight of 42 d broilers (*p* < 0.05). Moreover, there are positive relationships between *Intestinimonas* and thigh muscle rate, *Bifidobacterium* and abdominal fat rate, *Anarotruncus* and BW of 42 d broilers, *Escherichia-Shigella* and BW of 21 d broilers (*p* < 0.05).

## Discussion

The gene expression of TCA cycle-related enzymes was tested, under the assumption that intestinal energy homeostasis could be affected by dietary fat-to-fiber ratios. TCA cycle, also named the citrate cycle, is known as the center of cellular aerobic metabolism, and the metabolic enzymes of TCA cycle play important roles in regulating cellular and intestinal energy metabolism (Li et al., 2022; Luo P. et al., 2022). The catabolism of carbohydrate, protein and lipid produce acetyl-CoA, a two-carbon metabolite that could enter the TCA cycle to produce ATP. The up-regulated gene expression of enzymes related to TCA cycle including *OGDH*, *SDHB* and *SUCLG2* in broilers fed with an HF-LD diet probably indicated higher energy metabolism of the intestine. Additionally, functional analysis in microbiota also showed that citrate cycle increased in birds fed HF-LD diet. Fiber-rich diet would increase extra energy expenditure and reduce the energy used for intestinal proliferation and development (JøRgensen et al., 1996). Therefore, increasing soybean oil content and decreasing the proportion of NSP-rich ingredients could enhance gene expression of TCA cycle-related enzymes and increase the metabolic pathway of TCA cycle of microbiota, contributing to better energy homeostasis in the intestine.

The intestine was responsible for nutrient absorption and digestion and the intestine epithelium was central to preserving gut homeostasis. These characteristics indicated that the intestine highly depended on energy (Yilmaz et al., 2012). Birds fed HF-LD diet were more capable of maintaining a state of energy balance, presumably because less digestion and metabolic efforts were needed to utilize the diets (Noblet et al., 1994b; Li et al., 2017). Our results showed that HF-LD diet up-regulated gene expression of transporter proteins related to glucose (*SGLT1* and *GLUT5*), suggesting that high fat-low dietary fiber would enhance glucose absorption of broilers. Additionally, the starch source differs between wheat and corn, which may affect the nutrient transporter genes. In agreement with our study, Agyekum et al. (2015) demonstrated that high fiber diet decreased the active transporter genes of *GLUT*5 and the passive transporter genes of *SGLT1*. The metabolic pathways of microbiota including pentose phosphate, glycolysis/gluconeogenesis and pyruvate metabolism were closely correlated and interwoven into a network of energy metabolism (Zhang Y. et al., 2019). These metabolic pathways were enriched in HF-LD diets. On the contrary, dietary LF-HD decreased intestinal absorption and increased intestinal motility of broilers, producing heat energy loss in the intestine (Sanchez et al., 2021). It was well known that the intestinal tract was in contact with blood and thus circulating nutrients. Interestingly, the content of lipoprotein and triglyceride in serum increased in birds fed diet with LF-HD, accompanied by improvement of lipid metabolism (ether lipid, arachidonic acid and sphingolipid) of microbiota in hindgut. The reason for this improvement in lipid metabolism could be a compensatory response to a low-fat content in diet. Lipid metabolism was reported to be correlated with abdominal fat that was wasted as a slaughter product while carbohydrate metabolism participated in intramuscular fat deposition that was beneficial to growth performance (Luo N. et al., 2022). Consequently, it could be illustrated that dietary HF-LD increased gene expression of glucose transporters in the foregut and enhanced microbial metabolites related to carbohydrates in the hindgut.

Dietary energy density profoundly influences microbiota composition and abundance (Adewole and Akinyemi, 2021). Low energy density decreased the amount of nutrients available for bacterial fermentation, thereby reshaping bacterial structure (Attia et al., 2021). In addition, nutrient utilization of microbiota in hindgut is closely related to energy metabolism (Yamamura et al., 2021). We found that HF-LD diet improved the abundance and diversity of intestinal microbiota. In our study, the increasing fat-to-fiber ratios were accompanied by lower fiber content and NSP content. This was partly in consistent with studies that dietary high fiber or NSP content could enrich the abundance and diversity of microbiota and provide extra energy for animals (Zhao et al., 2023). Bacteroidotas are capable of hydrolyzing indigestible carbohydrates. The improvement of Bacteroidotas in HF-LD diet probably indicated an enhancement of carbohydrate metabolism. A higher abundance of the Firmicutes to Bacteroidotas was reported to increase the capacity to harvest energy from diets (Zhang Y. et al., 2019). In our study, HF-LD decreased the Firmicutes to Bacteroidotas ratio, probably due to the adequate energy available for broilers. Moreover, dietary HF-LD enriched the abundance of Proteobacteria to obtain energy from glycolysis, making up for the lack of energy obtained by Bacteroidotas (Zhang Y. et al., 2019). *Intestinimonas*, *Eubacterium* and *Lactobacillus* were enriched in HF-LD diet, which could ferment complex dietary carbohydrates to produce SCFA and provide energy for intestinal epithelial cells (Gong et al., 2021; Leyva-Diaz et al., 2021). More specifically, *Peptococcus*, as an NSP-degrading bacterium, could reduce the viscosity of intestinal contents and improve the nutrient value of diets, ultimately promoting energy utilization (Gao et al., 2022). NSP encapsulates other nutrients and hinders further absorption, increasing extra movement of intestine and contributing to the production of heat increment (Chen et al., 2023). In the current study, a high abundance of *Peptococcus* observed in HF-LD diet may accelerate the degradation of NSP and contribute to the reduction of heat increment. Therefore, HF-LD diet with appropriate high-fat content increased abundance of microbiota for energy harvesting and NSP-fermentation, thereby improving the energy status of intestine. Dietary high fat achieved its benefits on animals potentially by shifting microbiota toward propionate fermentation (Li et al., 2023). Further study should be conducted to explore the effects of fat and fiber content on the production of SCFA, particularly butyrate and propionate, which may provide information for the links between microbial changes and hindgut energy homeostasis. Therefore, HF-LD enriched microbiota diversity and provided better niches for the bacteria that could make better use of non-digestible carbohydrates.

These microbial changes in hindgut may bring about functional differences in metabolites. Our analysis using Tax4Fun found that dietary HF-LD was positively associated with carbohydrate metabolism, energy metabolism and some lipid metabolism. Glycolysis/gluconeogenesis, pyruvate metabolism and TCA cycle were enhanced in HF-LD diet, which may be the links between carbon metabolism and energy metabolism. On the one hand, pyruvate is a key intermediate of carbohydrate metabolism and participates in lipid and TCA cycle. Moreover, metabolites of fructose and mannose could enter glycolysis/gluconeogenesis and pyruvate metabolic pathways. On the other hand, the metabolic of pentose phosphate provides nicotinamide adenine dinucleotide phosphate oxidase (NADPH) for lipid metabolism, which links lipid and carbohydrate metabolism. More specifically, *Alistipes,* as a commensal microbiota, was known to be correlated with glycolysis/gluconeogenesis and pyruvate metabolic pathways, meanwhile a high abundance of *Alistipes* in an HF-LD diet could prove to be beneficial in carbohydrate metabolism (Zhang Y. et al., 2019). The complex oligosaccharides utilization and carbohydrate metabolism of Lactobacilli have been extensively studied (Zunga et al., 2021). Moreover, enzymes derived from Lactobacilli and Bifidobacteria play important roles in mediating the deconjugation of bile acid (Sun et al., 2019). There was evidence that the elevation of primary and secondary bile acid in HF-LD diet probably indicated an enhancement of intestinal energy metabolism (Hu et al., 2022). The detailed mechanism by which dietary fiber and fat interact with the gut microecology is still not yet well characterized. However, HF-LD diet appeared to increase microbiota diversity and restructure microbial composition as well as metabolic pathways such as glycolysis, gluconeogenesis and pyruvate metabolism.

The correlation analysis between differential microbiota and growth performance further demonstrated the beneficial effects of HF-LD diet. Consistent with our study, *Peptococcus*, *Lactobacillus* and *Intestinimonas* were reported to be positively related to body weight and feed intake (An et al., 2022; Zhang et al., 2022). Our study revealed harmful effects of *CHKCI001* that were negatively correlated with body weight of broilers. The lack of metabolic information for CHKCI001 hinders the interpretation for the improvement in the LF-HD diet. In agreement with our study, a fiber-rich diet could impair the feed intake of pigs in order to enhance energy digestibility (Schoenherr et al., 1989). Consequently, it was not surprising given that microbiota changes in response to a HF-LD diet were positively correlated with growth performance of broilers.

It is reasonably given that a high fat-to-fiber ratio reshaped composition and function of microbiota and improved energy metabolism in the small intestine, thereby contributing to profound intestine functions and improving the overall growth of broilers. The study provides a new perspective on optimization of dietary nutrition component targeting profound energy homeostasis and microbial functions, which is of great importance in optimizing growth performance of broilers and maximum productive benefits. Nevertheless, the underlying mechanism of dietary fat-to-fiber ratio on regulating microbiota and its metabolites of broilers should be further studied.

## Conclusion

Dietary HF-LD, formulating by increasing soybean oil and reducing NSP-rich ingredients, increased jejunal gene expression of glucose transporter and TCA cycle of broilers. Correspondingly, HF-LD diet enriched microbiota diversity and provided better niches for the bacteria, which could make better use of non-digestible carbohydrates. The microbial fermentation contributes to improving metabolic pathways including glycolysis/gluconeogenesis, pyruvate and TCA cycle. In addition, the growth performance was positively correlated with microbiota, which further confirmed the beneficial effects of the HF-LD diet. These findings indicate that dietary composition, particularly fat and fiber contents, should be considered for ensuring optimum intestinal health and overall growth in poultry.

## Data availability statement

The datasets presented in this study can be found in online repositories. The names of the repository/repositories and accession number(s) can be found at: https://www.ncbi.nlm.nih.gov/sra; PRJNA952536.

## Ethics statement

The animal studies were approved by Institutional Animal Care and Use Committee, China Agricultural University. The studies were conducted in accordance with the local legislation and institutional requirements. Written informed consent was obtained from the owners for the participation of their animals in this study.

## Author contributions

QJ: Conceptualization, Writing – review & editing, Data curation, Investigation, Writing – original draft. LZ: Writing – review & editing, Supervision. ZB: Writing – review & editing, Conceptualization, Investigation, Project administration. BZ: Conceptualization, Project administration, Writing – review & editing, Funding acquisition, Methodology, Supervision.

## Footnotes

^1^ https://ccb.jhu.edu/software/FLASH/index.shtml

^2^ http://www.drive5.com/uparse/

^3^ https://www.mothur.org/wiki/Download_mothur

^4^ http://qiime.org/install/index.html

^5^ http://picrust.github.io/picrust/
